# Identification of hub genes in heart failure by integrated bioinformatics analysis and machine learning

**DOI:** 10.3389/fcvm.2023.1332287

**Published:** 2024-01-05

**Authors:** Tengfei Wang, Yongyou Sun, Yingpeng Zhao, Jinhe Huang, Ying Huang

**Affiliations:** ^1^ Department of Cardiology, The First Affiliated Hospital of Anhui Medical University, Hefei, China; ^2^ Department of Cardiology, Funan County People’s Hospital, Fuyang, China

**Keywords:** heart failure, machine learning, cleaved-PARP1, PARP1, SDSL

## Abstract

**Objective:**

To screen feature genes of heart failure patients through machine learning methods, in order to identify characteristic genes driving heart failure and investigate the progression of heart failure

**Methods:**

Heart failure patient samples were downloaded from the public database GEO (Gene Expression Omnibus), including the datasets GSE116250, GSE120895, and GSE59867. GSE116250 and GSE120895 were used as the testing set, while GSE59867 was used as the validation set. LASSO regression analysis and SVM-RFE were utilized to identify feature genes.

**Results:**

Analysis showed that among the differentially expressed genes between normal and heart failure patients, 9 genes were upregulated and 10 genes were downregulated. ROC curve analysis in the training set showed that TAGLN and SGPP2 had AUC values greater than 0.7. Moreover, SDSL and SMTNL2 had even higher AUC values of greater than 0.9. However, further analysis in the validation set showed that only SDSL had an AUC value greater than 0.7. Western blot experiments, RT-PCR, and ISO-induced experiments confirmed that SDSL was highly expressed in heart failure patients and promoted heart failure progression. In addition, SDSL promoted PARP1 expression and knockdown of SDSL expression led to decreased Cleaved-PARP1 expression and reduced cardiomyocyte apoptosis. Conversely, overexpression of SDSL resulted in increased PARP1 expression and myocardial cell apoptosis. These results suggest that elevated expression of SDSL in cardiomyocytes from heart failure patients may be an important factor promoting the occurrence and development of heart failure.

**Conclusions:**

Using machine learning methods and experimental validation, it has been demonstrated that SDSL is a driving gene in patients with heart failure, providing a new treatment direction for clinical treatment.

## 1 Introduction

Heart failure (HF) remains one of the most common, complex, debilitating, and deadly diseases encountered by physicians in various medical fields (1). Heart failure is the leading cause of death in cardiovascular diseases. American Heart Association (AHA) defines it as a complex clinical syndrome that is the result of various structural or functional disorders of the heart that impair ventricular filling or ejection capacity. In most cases, it refers to a decrease in myocardial contractile function that prevents the ejection volume from meeting the metabolic needs of the body, insufficient blood perfusion in organs and tissues, and often accompanied by pulmonary and/or systemic circulation congestion. However, HF patients typically present with non-specific signs and symptoms and have a wide range of differential diagnoses, making the diagnosis and prognosis of HF challenging based solely on clinical manifestations. Given the increasing prevalence of HF worldwide, timely treatment and management of this potentially fatal disease through various pharmacological and/or non-pharmacological means is crucial for patients (2). With the development of high-throughput sequencing, our understanding of genetic diversity is rapidly advancing, providing enormous potential for the development of genetic biomarkers.

Biomarkers, such as genes or other genetic material related to disease susceptibility, can serve as novel diagnostic methods for HF. These biomarkers are becoming increasingly important in current medical practice as they provide a simple way to diagnose or monitor disease progression. In fact, biomarkers have been used to assess the condition of HF patients, mainly by evaluating the expression levels of certain genes in the patients (3, 4). Thus, biomarkers may be used to evaluate the effectiveness of therapeutic interventions in HF patients. Biomarkers also have the potential to serve as treatment targets for HF, helping to determine the best drugs for treatment (5). Currently, there are no relevant reports on the research of SDSL in cardiovascular diseases, and even in tumors, only a few studies have been reported. For example, there are few studies on the relationship between SDSL expression and poor prognosis in acute myeloid leukemia (6).

In this study, we used machine learning methods to identify characteristic genes of heart failure through public databases. As a result, we discovered that SDSL could promote the progression of heart failure, which provides a new therapeutic direction for heart failure treatment.

## 2 Materials and methods

### 2.1 Data collection

Three raw datasets (GSE51472 (Normal:14, Heart failure samples:50), GSE12644 (Normal:8, Heart failure samples:47), and GSE83453 (Normal:47, Heart failure samples:390)) including gene expression data for heart failure patients and controls were downloaded from the GEO (https://www.ncbi.nlm.nih.gov/geo/) database. The GSE116250 and GSE59867 datasets include 50 and 390 patients with preserved ejection fraction, respectively, while GSE120895 includes 47 patients with reduced ejection fraction heart failure. Supplementary Table 1 shows the distribution of heart failure patients in all datasets. GSE116250 and GSE120895 as the training set, and GSE59867 as the test set.

### 2.1 Data processing and differentially expressed gene screening

First, background calibration, normalization, and log2 transformation were performed on the three HF raw datasets using in R (4.1.2). When multiple probes identified the same gene,the average value was calculated to determine its expression.Following the merging of the two datasets, the Bioconductor “SVA” R package was applied to eliminate batch effects. Finally, |log2 Fold change (FC)|>2 and adjust *p*-value <0.05 were set as the criteria for identifying DEGs using Limma package (7).

### 2.3 Functional enrichment analysis

Gene Ontology (GO) studies, including biological processes, molecular functions, and cellular components, we utilized the “clusterprofiler” software package (8). Additionally, pathway enrichment analysis was performed using genes from the Kyoto Encyclopedia of Genes and Genomes (KEGG), with the threshold set at *p* < 0.05. To analyze the Disease Ontology (DO) enrichment, we used the “DOSE” software package and *p*-value <0.05, adjust *p*-value <0.05. The fundamental concept of Gene Set Enrichment Analysis (GSEA) is to rank genes based on their differential expression across two types of samples, using predefined genes and testing whether the set of predefined genes is enriched at the top or bottom of this ranking table. To perform GSEA enrichment analysis, we downloaded c2.cp.kegg.v7.4.symbols.gmt and c5.go.v7.4.symbols.gmt datasets from the GSEA database. The “clusterProfiler” package was utilized for this analysis.

### 2.4 Machine learing and ROC curve analysis

Two machine learning algorithms were used to further screen candidate genes for HF diagnosis. LASSO (Least Absolute Shrinkage and Selection Operator) analysis is a regression analysis method proposed by Robert Tibshirani. We conducted the lasso regression analysis using package “glmnet”. Support Vector Machine Recursive Feature Elimination (SVM-RFE) was initially proposed by Guyon et al. for classification of cancer using only two types of data in feature extraction. It is an embedded method. Utilized differential genes from the training set and performed SVM-RFE analysis using the “e1071”, “kernlab”, and “caret” packages. The intersection genes of LASSO and SVM-RFE were considered as candidate hub genes in HF diagnosis. Further, ROC curves were generated in the training and test sets to evaluate the model's effectiveness.

### 2.5 Western blot experiment

For Western blot, the main materials were RIPA lysis buffer (Biyuntian, p0013b), BCA protein concentration determination kit (Biyuntian, p0010) and PBS (Symantec). Antibodies were purchased from Abcam, and concentration dilution of SDSL (ab179435) 1:1,000, Cleaved-PARP1 (ab32064) 1:1,000, PARP1(ab191217) 1:1,000, GAPDH (#5174) purchased from CST, concentration dilution 1:20,000. After the cells were cultured in 6-well plates, 150 µl of lysis buffer was added to each well, immediately scrape and collect cells into a new 1.5 ml tube (complete on ice), and protein concentration in the lysate was quantified with the BCA kit. The cell lysates were loaded onto SDA-PAGE gels and separated by electrophoresis. The protein bands were transferred onto PVDF membranes, and performing development exposure. The ISO induction experiment is divided into five groups, including 10 um, 25 um, 50 um, and 100 um. SDSL interference experiment divide into four groups, including AC16, siRNA-NC (40 nm, 24 h), SDSL siRNA (40 nm, 24 h), and ISO (50 um, 24 h). The experiment was conducted in triplicate and repeated three times.

### 2.6 Real-time quantitative PCR (qPCR)

Real-time quantitative PCR (qPCR) is a method used in DNA amplification reactions to measure the total amount of product after each cycle of polymerase chain reaction (PCR) using fluorescent chemistry. Firstly, culture AC16 cells, then add 1 ml of Trizol to the cells and lyse them into a 1.5 ml EP tube, add 200 ul of chloroform, gently invert several times, mix well, leave at room temperature for 5 min, centrifuge at 12,000 rpm, 4°C for 15 min, and measure RNA concentration using a spectrophotometer. The expression levels of SDSL mRNA were detected by qPCR. The primer sequences of GAPDH and SDSL were, respectively, h-GAPDH-F primer sequence 5'→3': ACAACTTTGGTATCGTGGAAGG, h-GAPDH-R primer sequence 5'→3': GCCATCACGCCACAGTTTC, h-SDSL-F primer sequence Sequence 5'→3': GACGGCTGGGAGAATGTCC, h-SDSL-R primer sequence 5'→3': ATGGCCGCATTGAAGCAGT. The ISO induction experiment is divided into five groups, including 10 um, 25 um, 50 um, and 100 um. Repeat each experiment three times.

### 2.7 Ac16 cell apoptosis experiment

Apoptosis is one of the fundamental characteristics of cells, playing a crucial role in embryonic development, tissue repair, and stability of the internal environment in the body. The steps of the apoptosis experiment include: (1) After digestion with trypsin without EDTA, cells were collected by centrifugation at 4°C for 5 min at 300 g. The digestion time of pancreatic enzymes should not be too long to prevent false positives. (2) Preparation 1 × Binding Buffer: Dilute 4 times with deionized water × Binding Buffer (4 ml binding buffer + 12 ml deionized water). (3) Wash the cells twice with PBS pre cooled at 4°C, each time requiring 300 g, and centrifuge at 4°C for 5 min. (4) Add 250 μl 1× Binding Buffer resuspended cells and adjusted their concentration to 1 × 106 cells/ml. (5) Take 100 μ Transfer the cell suspension into a 5 ml flow cytometry tube and add 5 μ Annex FITC and 10 μ PI, gently mix well. (6) Avoid light and react at room temperature for 15 min. (7) Join 400 μl 1× Binding Buffer, mix well, and test the sample within 1 h. ISO induced apoptosis of AC16 cells was divided into five groups, including 10 um, 25 um, 50 um,and 100 um, and divide AC16 cell apoptosis induced by ISO interference with SDSL expression into four groups, including control, SDSL siRNA, ISO, SDSL siRNA + ISO.

### 2.8 SDSL overexpression

To investigate the impact of SDSL overexpression on apoptosis in AC16 cells, we performed overexpression experiments using SDSL plasmids. The overexpression sequence of SDSL was as follows: SDSL-F AGCGATTCGCCACCATGGGGGGAGCCTCTGCGAGA and SDSL-R TTTGTAGTCGGATCCCTGCAGTTCAGCTGTGTGTTTT. Subsequently, Western blot, Real time quantification, and apoptosis experiments were performed on AC16 cells overexpressing SDSL. The experimental groups were divided into four subgroups, and each experiment was replicated three times to ensure robustness and reliability of the results.

### 2.9 Statistical analysis

All statistical analyses were performed by R 4.1.2, *p* values <0.05 were considered statistically significant. Related R packages including “sva”, “limma”, “pheatmap” and “glmnet”. and other related R packages were downloaded from Bioconductor packages or R packages. For each analysis, statistical significance was set at *p*-value <0.05.

## 3 Result

### 3.1 Identification of candidate heart failure related differential gene

**Figure 1:**
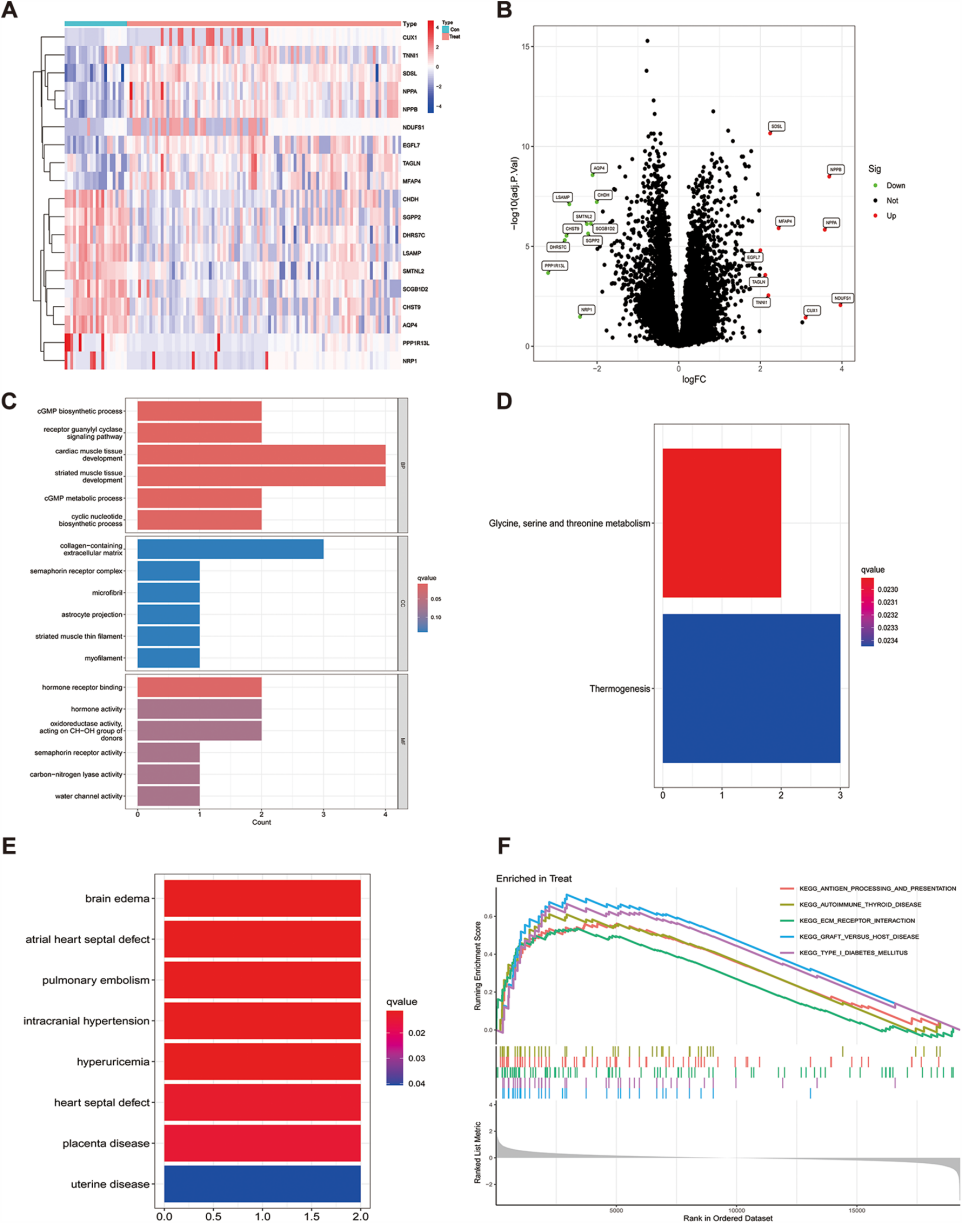
Differential gene heatmap and gene enrichment analysis, (**A**) indicates the differential gene heat map after merging the datasets, (**B**) indicates the differential gene volcano heat map after merging the datasets, (**C**) GO enrichment analysis for each differential gene, (**D**) KEGG enrichment analysis for each differential gene, (**E**) enrichment analysis for differential genes in DO, (**F)** expresses GSEA enrichment analysis for genes in the tumor group.

The R software was used to normalize the GSE116250 and GSE120895 training sets, and a total of 19 genes with differential expression were obtained by differential analysis, of which 10 genes were down-regulated and 9 genes were up-regulated, as shown in Table 1 and (Figure 1A), and the genes with significant differential expression were annotated by volcano plots (Figure 1B).

**Table 1:** Differential gene expression.

id	logFC	AveExpr	*t*	*P*. Value	adj. *P*. Val	B
SDSL	2.239475	5.31462	8.887828	7.99 × 10^−15^	2.20 × 10^−11^	23.21108
AQP4	−2.11099	3.186507	−7.57735	8.44 × 10^−12^	2.70 × 10^−09^	16.49716
NPPB	3.68695	9.061614	7.524013	1.11 × 10^−11^	3.20 × 10^−09^	16.23042
CHDH	−2.00698	1.947702	−6.74885	5.75 × 10^−10^	6.02 × 10^−08^	12.43361
LSAMP	−2.67948	2.973226	−6.68218	8.00 × 10^−10^	7.80 × 10^−08^	12.11493
SCGB1D2	−2.15224	2.257207	−6.10948	1.30 × 10^−08^	7.31 × 10^−07^	9.438457
SMTNL2	−2.24937	4.540269	−6.10128	1.35 × 10^−08^	7.54 × 10^−07^	9.400955
MFAP4	2.447268	6.16297	5.967751	2.55 × 10^−08^	1.24 × 10^−06^	8.794525
NPPA	3.577934	10.46512	5.923948	3.13 × 10^−08^	1.46 × 10^−06^	8.597121
SGPP2	−2.2203	2.667199	−5.80425	5.47 × 10^−08^	2.29 × 10^−06^	8.061666
CHST9	−2.74878	2.902939	−5.73782	7.44 × 10^−08^	2.92 × 10^−06^	7.767104
DHRS7C	−2.79079	5.214339	−5.60007	1.40 × 10^−07^	4.91 × 10^−06^	7.16234
EGFL7	2.000391	5.251084	5.276298	6.00 × 10^−07^	1.59 × 10^−05^	5.77477
PPP1R13l	−3.20057	8.850626	−4.49841	1.60 × 10^−05^	0.000213	2.658742
TAGLN	2.121351	7.16273	4.424147	2.16 × 10^−05^	0.000271	2.379004
TNNI1	2.196924	4.478899	3.650588	0.00039	0.002852	−0.32736
NDUFS1	3.962628	13.81949	3.238772	0.001556	0.00876	−1.60004
NRP1	−2.41843	9.043121	−2.67862	0.008439	0.03368	−3.12349
CUX1	3.108471	9.434677	2.63947	0.009416	0.036648	−3.22054

### 3.2 Functional enrichment analysis

To explore the potential biological functions of the 19 differential genes summarized in the previous work, we performed GO analysis and KEGG pathway enrichment analysis. In biological process (BP), it was significantly (*p* < 0.05) associated mainly with regulation of anatomical size, regulation of tubular diameter, and maintenance of vascular diameter, and in molecular function (MF), it was significantly associated with molecular functions such as extracellular matrix, contractile fibers, and astrocyte projection, and we also enriched cellular component (CC) functions, which were significantly associated with hormone activity, hormone receptor binding, and signaling hormone receptor activity and signaling receptor activity(Figure 1C). In KEGG pathway enrichment analysis, it was mainly associated with glycine, serine and threonine metabolism and thermogenesis pathways (Figure 1D), and in DO enrichment analysis, 19 differential genes were shown to be mainly associated with diseases such as atrial septal defect, pulmonary embolism, and septal defect (Figure 1E).

To analyze how genes cause the developmental process of heart failure, we performed GSEA enrichment analysis on the GSE116250 and GSE120895 training sets. The results showed that differential gene expression in pathway enrichment in heart failure samples was significantly associated with KEGG_ANTIGEN_PROCESSING_AND_PRESENTATION, KEGG_ECM_RECEPTOR_INTERACTION, KEGG_FOCAL_ADHESION, KEGG_GRAFT_VERSUS_HOST_DISEASE, and KEGG_TYPE_I_DIABETES_MELLITUS. In the normal sample it is mainly significantly associated with KEGG_INSULIN_SIGNALING_PATHWAY, KEGG_LONG_TERM_POTENTIATION and KEGG_PYRUVATE_METABOLISM (Figure 1F).

### 3.3 Identification of hub gene by lasso regression analysis and SVM-RFE analysis

**Figure 2:**
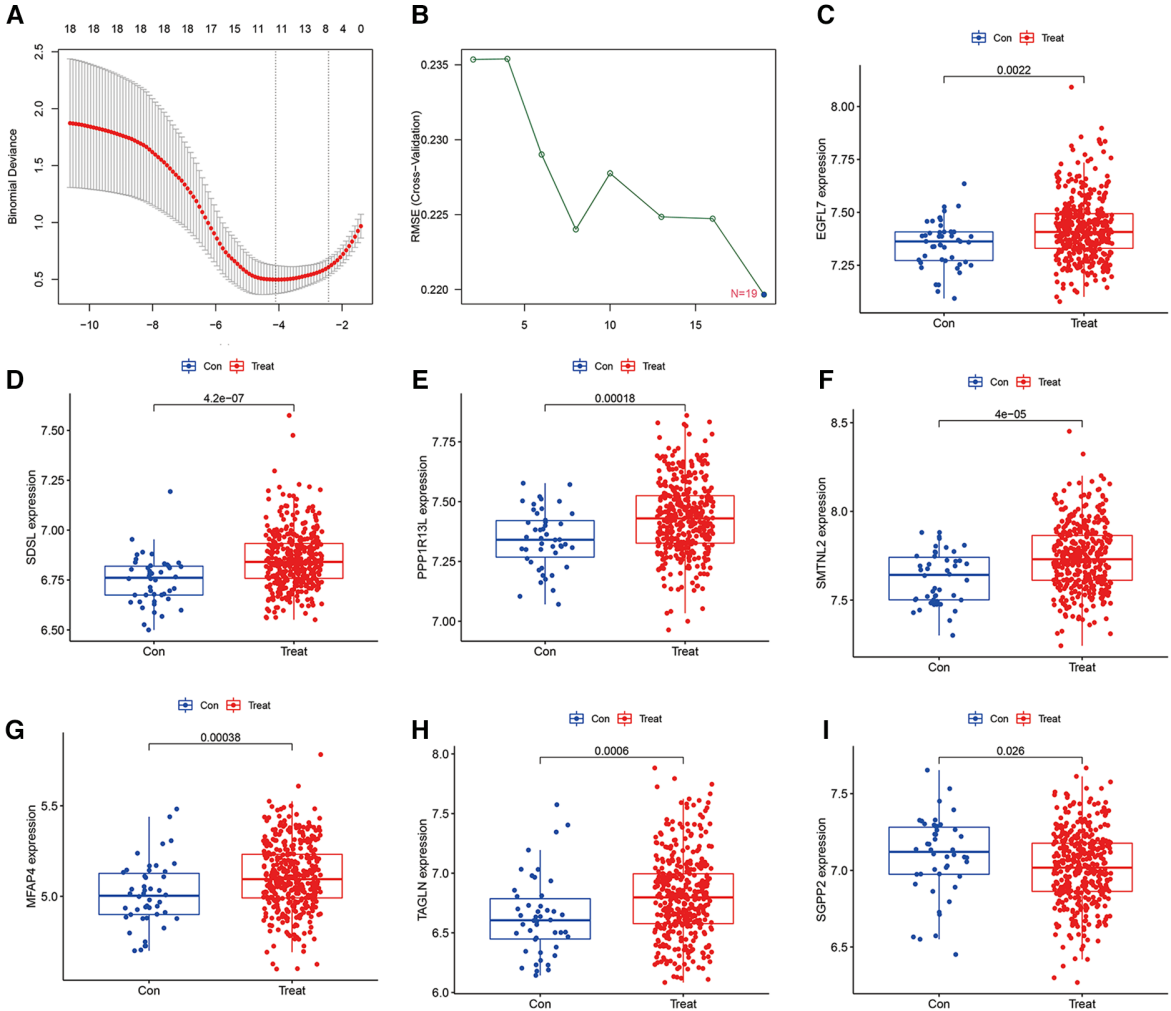
Machine learning screening of model hub genes and differential expression of candidate model genes in normal vs. abnormal tissues, (**A**) indicates 11 target genes were screened by LASSO regression analysis, (**B**) 19 model genes were screened by SVM-RFE method, (**C–H**) Showed that EGFL7, SDSL, PPP1R13l, SMTNL2, MFAP4 and TAGLN were highly expressed in heart failure patients, (**I**) indicated that SGPP2 was highly expressed in normal patients.

To detect heart failure hub gene, we screened disease genes by machine learning Lasso regression analysis and SVM-REF analysis on GSE116250 and GSE120895 training set heart failure samples, where lasso analysis screened to obtain 11 signature genes (Figure 2A), while SVM-REF obtained 19 signature genes by screening (Figure 2B), and 11 overlapping genes were obtained by taking the intersection of the two. The 11 genes were SDSL, AQP4, SCGB1D2, SMTNL2, MFAP4, SGPP2, EGFL7, PPP1R13l, TAGLN, NDUFS1, and NRP1. Furthermore, the 11 hub gene obtained by lasso and SVM-REF intersection were differentially expressed in normal and heart failure patients, of which 7 genes were significantly differentially expressed in the test set GSE59867. EGFL7, SDSL, PPP1R13l, SMTNL2, MFAP4 and TAGLN were highly expressed in heart failure samples (Figures 2C–H), and the high expression of these 6 hub gene may promote the progression of heart failure and be a risk factor for heart failure patients, while SGPP2 was highly expressed in normal samples (Figure 2I), which may play a protective role for heart failure patients.

**Figure 3:**
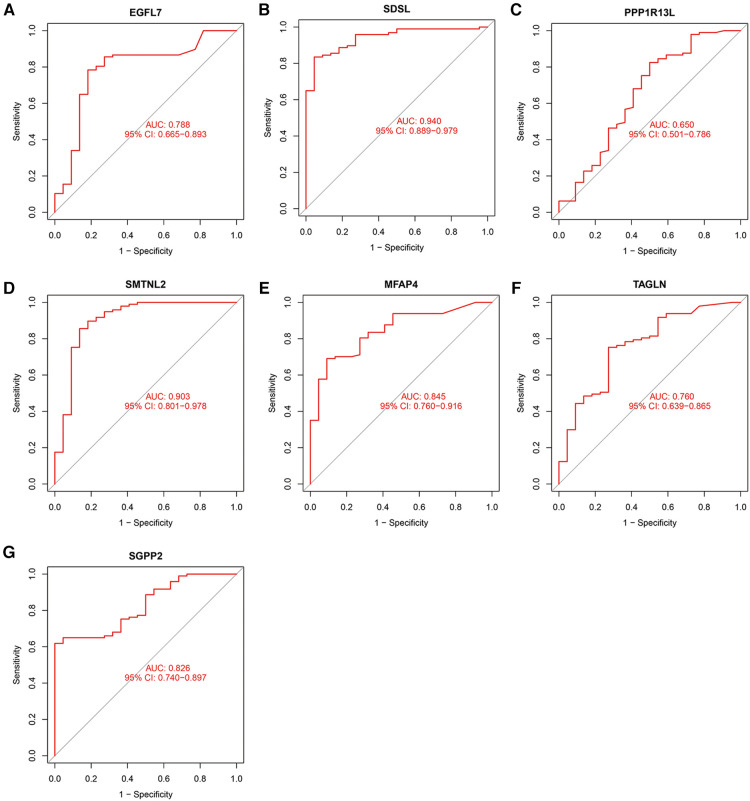
Candidate model marker genes (**A**) EGFL7, (**B**) SDSL, (**C**) PPP1R13l, (**D**) SMTNL2, (**E**) MFAP4, (**F**) TAGLN and (**G**) SGPP2 in the test dataset for ROC model validation.

**Figure 4:**
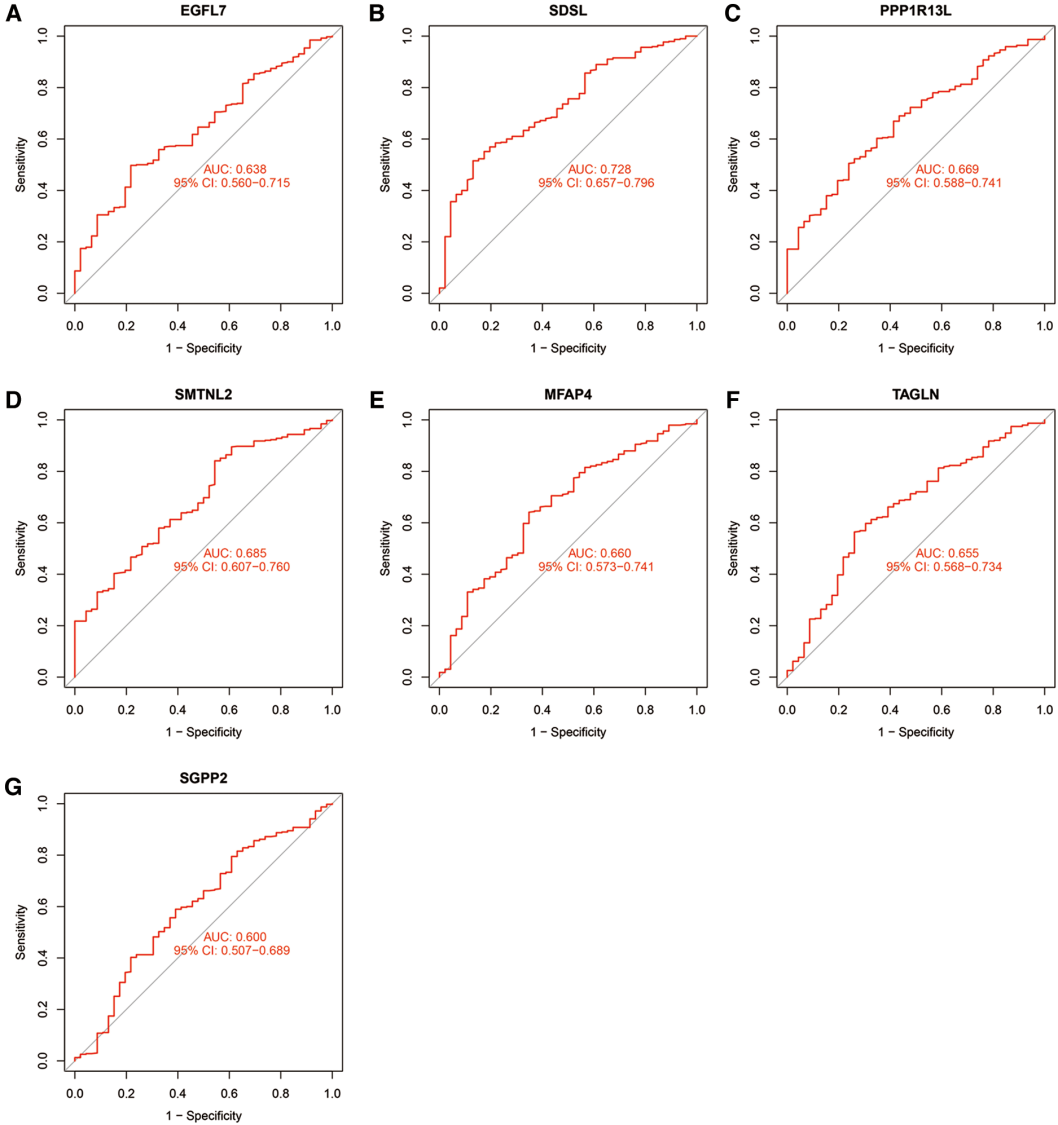
Candidate model marker genes (**A**) EGFL7, (**B**) SDSL, (**C**) PPP1R13l, (**D**) SMTNL2, (**E**) MFAP4, (**F**) TAGLN and (**G**) SGPP2 in the validation dataset for ROC model validation.

The accuracy of the seven model genes was further verified by ROC curves. In the training set, EGFL7, SDSL, SMTNL2, MFAP4, TAGLN and SGPP2 showed AUC curve areas greater than 0.7, while the AUC values of SDSL and SMTNL2 were even greater than 0.9 with high accuracy (Figure 3). However, only SDSL showed an AUC value greater than 0.7 in the test set, whereas EGFL7, SMTNL2, MFAP4, TAGLN, SGPP2 and SMTNL2 showed AUC values greater than 0.5, so SDSL may be a high risk factor in patients with heart failure (Figure 4).

### 3.4 Induction of apoptosis in Ac16 by ISO treatment and upregulation of SDSL expression

**Figure 5:**
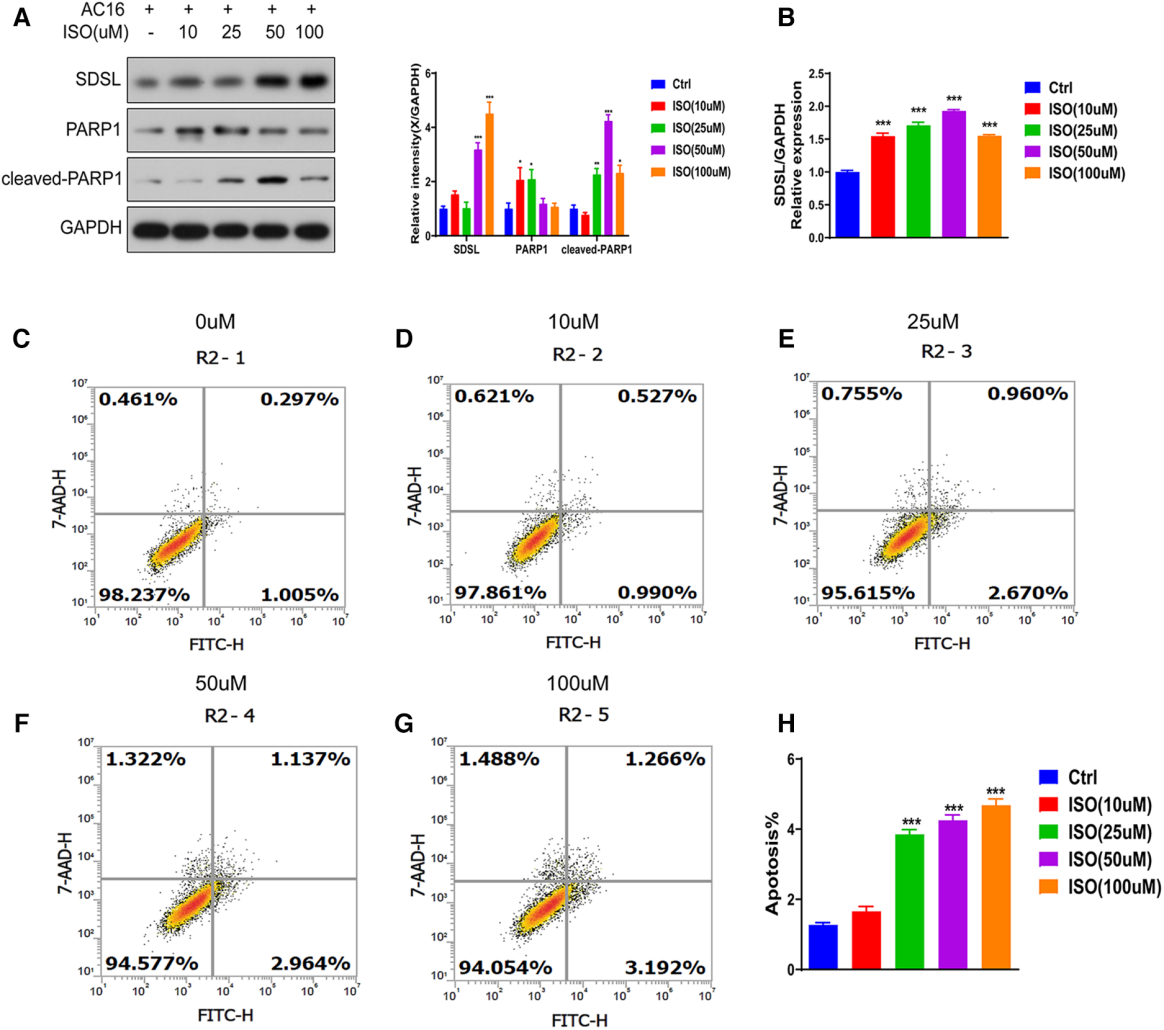
SDSL and PARP1 expression in heart failure cells. (**A**) Protein expression of SDSL and PARP1 in AC16 cells induced by ISO at different concentrations, (**B**) Protein expression of SDSL at different concentrations of ISO detected by Real-timePCR, (**C-H**) AC16 at ISO 10 um, 25 um, 50 um, 100 um induced apoptosis characteristics. *<0.05, **<0.01, ***<0.001.

Different concentrations of isoproterenol (ISO) can lead to increased expression of the protein SDSL. The expression of SDSL gradually increased in response to ISO concentrations of 0, 10, 25, 50, and 100 um (Figures 5A,B), with maximum expression observed at an ISO concentration of 100 um. Concurrently, the expression of PARP1 and Cleaved-PARP1 also increased with increasing ISO concentration (Figure 5A). Additionally, ISO induction resulted in significant apoptosis of cardiomyocytes in AC16 cells. Cell apoptosis gradually increased in response to ISO concentrations of 0, 10, 25, 50, and 100 um , with the most significant level observed at an ISO concentration of 100 um (Figures 5C–H).

### 3.5 Down-regulation of SDSL suppresses ISO-induced apoptosis in Ac16

**Figure 6:**
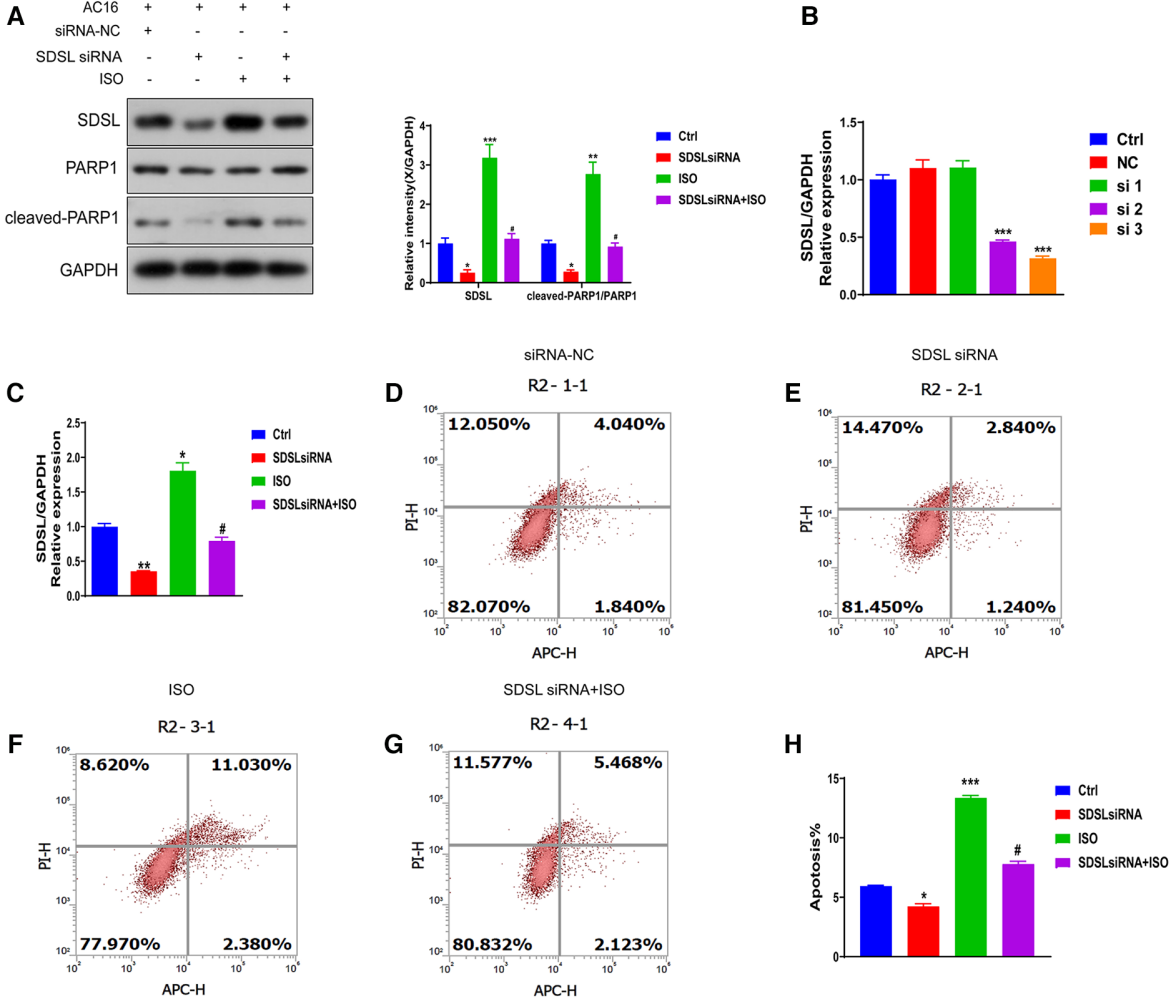
Effect of inhibition of SDSL expression on apoptosis. (**A**) The expression of SDSL and cleaved-PARP1 protein decreased after siRNA interference with SDSL. (**B**) selected the optimal sequence siRNA 3 for transfection of AC16 by interfering with the sequence. (**C**) The expression of SDSL was significantly decreased after transfection of SDSL compared with the control group, and increased after ISO-induced transfection. D-H SDSL induced apoptosis in cardiomyocytes after transfection. The ability of SDSL to induce apoptosis was decreased after (**D-H**) transfection, and SDSL after ISO induction promoted apoptosis in cardiac myocytes.

To investigate whether SDSL is a biomarker for heart failure, we downregulated its expression (Figures 6A,B) and observed a decrease in the expression of Cleaved-PRAP1. This resulted in reduced myocardial cell apoptosis compared to the normal group, indicating that inhibiting SDSL expression can regulate PRAP1 and prevent myocardial cell apoptosis. On the other hand, under ISO induction, downregulated SDSL expression increased myocardial cell apoptosis by regulating Cleaved-PRAP1 expression (Figures 6C–H). However, compared to the ISO group, the promotion of myocardial cell apoptosis decreased, which suggests that SDSL may be a targeted biomarker for heart failure, as it can regulate PRAP1 and inhibit ISO-induced myocardial cell apoptosis.

### 3.6 The overexpression of SDSL impacts the PARP1/cleaved-PARP1 expression and myocardial cell apoptosis

**Figure 7:**
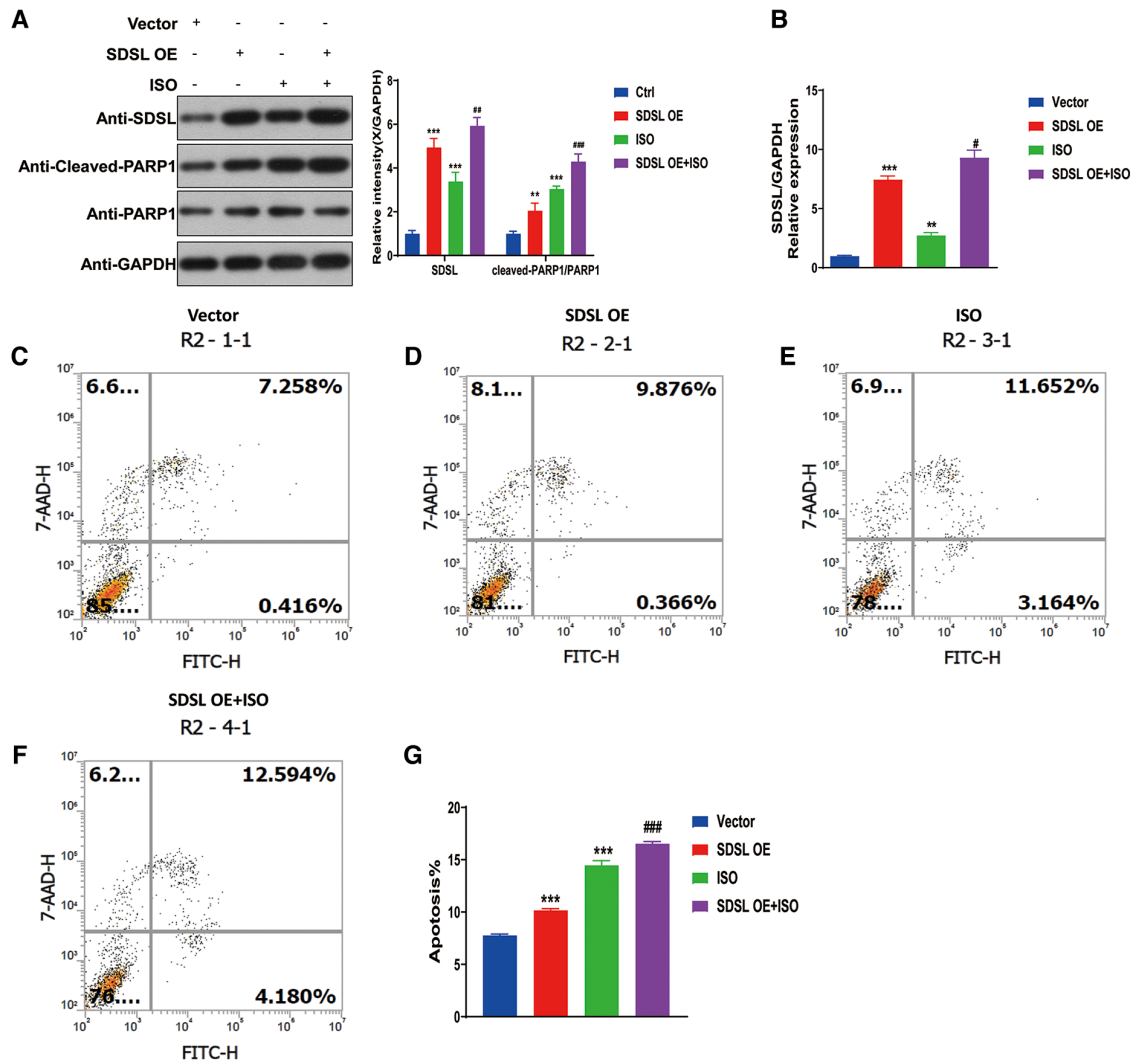
Overexpression of SDSL promotes PARP1/cleaved-PARP1 expression and cardiomyocyte apoptosis. (**A**) Weston blot results showed that overexpression of SDSL significantly promoted PARP1/cleaved-PARP1 expression. (**B**) RT-PCR reveals SDSL overexpression. (**C–G**) SDSL overexpression promotes cardiomyocyte apoptosis and promotes further apoptosis when ISO is added.

To investigate the impact of SDSL overexpression on PARP1/cleaved-PARP1 expression and myocardial cell apoptosis, the results of Western blot analyses revealed that SDSL overexpression led to increased expression of PARP1/cleaved-PARP1. Furthermore, when ISO was added to the system, the expression of SDSL and PARP1/cleaved-PARP1 increased (Figures 7A,B). These findings suggest that ISO stimulated AC16 to enhance the expression of SDSL and PARP1/cleaved-PARP1. With regards to apoptosis, SDSL overexpression was observed to promote myocardial cell apoptosis. Furthermore, ISO treatment in combination with SDSL overexpression led to increased apoptosis of myocardial cells (Figures 7C–G). Thus, it appears that SDSL acts as a factor that can promote myocardial cell apoptosis and contribute to the development of heart failure.

## 4 Discussion

Heart failure is a condition caused by the incapacity of the heart to pump blood efficiently, or to fill with blood, and has become common due to the variety of factors that cause it. In the United States alone, approximately 5.8 million people suffer from heart failure (9), with an associated mortality rate of 87.9 deaths per 100,000 people (10). The diagnosis of heart failure in patients is conventionally based on clinical examinations, medical history, physical evaluations, and chest radiographs. However, when used in isolation, these methods are inadequate for accurately diagnosing heart failure (11–13). Recent years have seen the advent of laboratory and imaging-based diagnostic criteria that provide accurate heart failure diagnosis (14, 15).Research into various biomarkers associated with heart failure pathophysiology is gaining momentum, with natriuretic peptide (16–18) and troponin (19, 20) standing out as promising markers. Though heart failure patient survival rates have grown, their mortality rates remain stubbornly high (21). Therefore, accelerating and perfecting the diagnosis of heart failure has become of paramount importance to heart failure patients.

Biomarkers are useful in understanding disease prediction, diagnosis, progression, degeneration, causation, or treatment outcomes. They can be cellular, biochemical, or molecular variations that can be detected in biological media, such as human tissues, fluids, or cells. BNP and NT-proBNP have emerged as popular biomarkers for heart failure (22). With most research studies associating the development of heart failure with their presence (23–26). However, given the plurality of factors that lead to heart failure, it is essential to identify new diagnostic markers for heart failure. This paper explores the genetic regulation of HF and its involvement in the pathological process, identifying biomarkers for the diagnosis and prognosis of HF.

This study identified a total of 11 differential genes in the training set, out of which 7 differentially expressed genes were analyzed further in the test set. The analysis revealed that heart failure samples had significantly higher expression levels of EGFL7, SDSL, PPP1R13l, SMTNL2, MFAP4, and TAGLN genes, which may promote the development of heart failure. Conversely, SGPP2 was highly expressed in normal samples and may serve as a protective gene against heart failure. The ROC model confirmed the accuracy of the identified genes. However, it is worth noting that SDSL was consistently significant in both the training and test sets, indicating its vital role in heart failure development.

SDSL is an enzyme with serine dehydratase-like activity that is primarily found in the liver (27). A gene similar to SDH has been identified through human genome sequencing (28), and this gene has been identified in human cancer cell lines, including those from lung, kidney, and brain cancer (29). However, there are only a few reports on the role of serine dehydratase-like (SDSL) in tumors and heart failure. In this study, we discovered that SDSL was upregulated in heart failure via machine learning techniques, High expression of SDSL promotes the development of heart failure. which was subsequently corroborated by experiments. Isoproterenol (ISO) can trigger apoptosis in cardiomyocytes. The inhibition of SDSL expression reduced ISO-induced cardiomyocyte apoptosis, whereas the promotion of SDSL expression elevated ISO-induced cardiomyocyte apoptosis. To examine the mechanism underlying the promotion of cardiomyocyte apoptosis by SDSL, we manipulated SDSL expression levels and discovered that PARP1 exhibited similar changes in expression. Therefore, we suggested that ISO induced SDSL expression promotes cardiomyocyte apoptosis by regulating PARP1, thereby promoting the progress of heart failure, and confirms that SDSL is a factor promoting heart failure. Although there is currently limited research on the role of SDSL in heart failure, our research fills this gap and provides new directions for the research and treatment of heart failure.

This study employed machine learning methods to identify characteristic genes associated with heart failure. Seven of these genes were identified as potential diagnostic markers for heart failure. Moreover, the study revealed that SDSL, via the regulation of PARP1 under ISO induction, plays a pivotal role in promoting the development and progression of heart failure in patients. This discovery highlights a novel research avenue for the diagnosis and treatment of heart failure.

## Data Availability

The datasets presented in this study can be found in online repositories. The names of the repository/repositories and accession number(s) can be found below: Gene Expression Omnibus, GSE116250, GSE120895, and GSE59867.
